# Are Ethnic Disparities in HbA1c Levels Explained by Mental Wellbeing? Analysis of Population-Based Data from the Health Survey for England

**DOI:** 10.1007/s40615-017-0346-0

**Published:** 2017-03-09

**Authors:** Kanayo Umeh

**Affiliations:** School of Natural Sciences & Psychology, Liverpool John Moores University, Liverpool, L3 3AF UK

**Keywords:** Ethnicity, HbA1c, Mental wellbeing, Energy

## Abstract

**Aims:**

It is unclear how ethnic differences in HbA_1c_ levels are affected by individual variations in mental wellbeing. Thus, the aim of this study was to assess the extent to which HbA_1c_ disparities between Caucasian and South Asian adults are mediated by various aspects of positive psychological functioning.

**Methods:**

Data from the 2014 *Health Survey for England* was analysed using bootstrapping methods. A total of 3894 UK residents with HbA_1c_ data were eligible to participate. Mental wellbeing was assessed using the Warwick-Edinburgh Mental Well-being Scale. To reduce bias BMI, blood pressure, diabetes status, and other factors were treated as covariates.

**Results:**

Ethnicity directly predicted blood sugar control (unadjusted coefficient −2.15; 95% CI −3.64, −0.67), with Caucasians generating lower average HbA_1c_ levels (37.68 mmol/mol (5.6%)) compared to South Asians (39.87 mmol/mol (5.8%)). This association was mediated by positive mental wellbeing, specifically concerning perceived vigour (unadjusted effect 0.30; 95% CI 0.13, 0.58): South Asians felt more energetic than Caucasians (unadjusted coefficient −0.32; 95% CI −0.49, −0.16), and greater perceived energy predicted lower HbA_1c_ levels (unadjusted coefficient −0.92; 95% CI −1.29, −0.55). This mediator effect accounted for just over 14% of the HbA_1c_ variance and was negated after adjusting for BMI.

**Conclusions:**

Caucasian experience better HbA_1c_ levels compared with their South Asian counterparts. However, this association is partly confounded by individual differences in perceived energy levels, which is implicated in better glycaemic control, and appears to serve a protective function in South Asians.

## Background

Poor glycaemic control is a major threat to public health [1, 2]. With steadily rising diabetes prevalence and incidence rates [3], the cost of hospitalisation related to poor glycaemic control is an increasing concern [4]. For example, in the UK, diabetes mellitus currently costs the health service over £10 billion annually [4]. A reduction in the length of hospital admission of just 0.61 days can save a health organisation over £400,000 [4]. Hospitalisation typically results from short and/or long-term complications associated with poor blood sugar (i.e. glycaemic) control [5]. Thus, improving blood sugar control can significantly reduce the costs of diabetes-related care [1], reducing the economic burden of the disease [4].

People from ethnic minority groups are at greater risk of elevated blood sugar levels [6, 7]. South Asians (e.g. Indian, Pakistani, Bangladeshi) in particular are 2.6 times more likely to be admitted to hospital for diabetes, compared to Caucasians [8]. The excess hospitalisation risk in South Asian patients may denote higher complication rates resulting from poor blood sugar control [1, 5, 9–11]. Although some evidence suggests no ethnic differences in blood glucose levels [12], other research indicates poorer glycaemic control amongst South Asian patients relative to Caucasians [13]. Thus, blood sugar control is especially important for South Asian patients [14].

Average blood sugar control over time is gauged using HbA_1c_ (glycated haemoglobin) test [5]. This test gives an indication of blood sugar concentration over the previous 2–3 months [15]. Test results are calibrated in percentages (values ≤6.5% indicating good blood sugar control), or millimoles per mole (values ≤48 mmol/mol denoting adequate glycaemic control). HbA_1c_ values of 6–6.4% (42–47 mmol/mol) indicates *pre-diabetes*, meaning a high risk of developing diabetes, while values ≥48 mmol/mol may denote *diabetes* [16]. A normal non-diabetic HbA_1c_ value is <36 mmol/mol (5.5%). Pre-diabetes is a clinically relevant state, as patients with this condition have an elevated risk of developing microvascular and macrovascular morbidities (e.g. coronary disease), and also type 2 diabetes [17]. Lifestyle and pharmacological interventions targeting pre-diabetes cases can help delay the onset of type 2 diabetes [17].

Lowering HbA_1c_ levels reduces the risk of long-term health problems, notably diabetes-related complications [1]. In many western countries, people diagnosed with pre-diabetes or diabetes are expected to undergo a HbA_1c_ test regularly (e.g. once or twice a year) [15]. The World Health Organisation (WHO) has recommended that the HbA_1c_ can also be used to diagnose type 2 diabetes in otherwise healthy people who are not currently diagnosed with diabetes or pre-diabetes [16, 18, 19].

A number of studies have compared HbA_1c_ outcomes in Caucasian and South Asian patients [12, 20–25]. For example, one study assessed HbA_1c_ outcomes in Caucasian and South Asian young adults attending a diabetes clinic [12]. While HbA_1c_ levels improved several years following diagnosis, they found no ethnic differences in glycaemic control. Another investigation assessed HbA_1c_ in South Asian and Caucasian women with a history of gestational diabetes and also found similar HbA_1c_ values across the groups [20]. However, an investigation of ethnic differences in HbA_1c_ outcomes amongst adults with normal glucose tolerance found higher HbA_1c_ levels in the South Asian participants (6.11 ± 0.58%) compared to their Caucasian counterparts (5.90 ± 0.40%) [21]. Another study also found a significant HbA_1c_ excess in South Asian patients, compared with Caucasians [22], partly attributing the differential to physiological mechanisms. Research on HbA_1c_ levels in children also show higher levels in South Asians, compared with Caucasians [23].

It is unclear to what extent *ethnic* differences in blood sugar control can be explained by variations in mental wellbeing. Some research has examined the association between psychological wellbeing and HbA_1c_ outcomes, with mixed results [24, 26–28]. One study assessed the relationship between anxiety and HbA_1c_ levels in South Asians and Caucasians and found no association [24]. Overall, however, none of the studies cited above, or others reported in the literature [25], have specifically examined the extent to which ethnic disparities in HbA_1c_ values are affected by *positive* psychological dispositions, such as decisiveness, feelings of self-worth, or empathy for others [29]. Poor mental wellbeing is associated with poorer diabetes self-management behaviours, including poor dietary habits, physical inactivity, and insufficient blood sugar monitoring [30]. By contrast, positive mental wellbeing (e.g. a positive emotional state) has been implicated in reduced diabetes risk [31]. One study found that diabetes patients exposed to group-based sessions promoting positive concepts such as empowerment and peer support reported significant HbA1c improvements after 1 year [32]. There has been limited research on ethnic differences in mental wellbeing. Some research indicates significant ethnic differentials, with Caucasians reporting higher levels of mental distress compared to South Asians, particularly in the context of diabetes [33]. However, other research suggests otherwise [34]. Given ethnic differences in HbA1c [21], and associations of these variables with mental wellbeing [31, 33], it is plausible psychological functioning partly explains HbA1c disparities across South Asians and Caucasians reported in the literature [22].

There are several important covariates to consider in this context. Suffering from long-term health conditions (e.g. diabetes, cardiovascular disease, cancer) may affect HbA_1c_, depending on the number and severity of diagnosed condition(s). Risk factors implicated in diabetes and cardiovascular disease (e.g. obesity, high blood pressure) may vary across ethnic groups [6], or be associated with blood sugar levels [35]. For example, BMI (body mass index) has been found to be associated with HbA_1c_ data [36, 37]. Socioeconomic deprivation has been associated with diabetes complications and blood sugar control [38]. Deprivation also impacts differentially across ethnic groups, with an increased diabetes-related hospital readmission risk for Caucasians (by contrast, the risk of readmission in South Asians is lower, compared with Caucasians) [8]. Thus, while South Asian diabetes patients are more likely to be hospitalised for diabetes-related complications, Caucasians seem more susceptible to the effects of deprivation, albeit the implications for blood sugar control are less clear-cut [39].

Overall, there is a need to better understand the confounding effect of positive psychological states on ethnic differences in HbA_1c_ levels. Hitherto, no study has been found specifically investigating this topic. Thus, the investigation reported here aimed to address this gap in the literature, while adjusting for important covariates. Given evidence suggesting ethnic disparities in HbA_1c_, whereby South Asians show poorer outcomes [21], and given evidence associating mental wellbeing with both ethnicity [33, 34], and diabetes risk [31], it was expected that (a) ethnicity will predict HbA_1c_ outcomes, with South Asians showing higher HbA_1c_ levels, and (b) the association between ethnicity and HbA_1c_ levels will be mediated by individual differences in positive mental wellbeing.

## Methodology

### Participants and Procedure

This study analysed data from the 2014 *Health Survey for England* [40]. The HSE has been conducted since 1994 and monitors health conditions and other related data (e.g. lifestyle, blood pressure, weight and height, mental wellbeing, and health service use) in UK households. Members of a household first complete a questionnaire booklet. This is followed shortly thereafter by a nurse visit, during which additional (primarily biomedical) data is collected from participants. The 2014 survey obtained data from 10,080 adults (aged 16 years and over) and children (aged up to 15 years). Data on blood sugar control (HbA_1c_ mmol/mol results) was specifically relevant to the present study, regardless of diabetes status. A total of 3894 respondents provided valid HbA_1c_ data. These cases, together consisted of 1987 (53.5%) women and 1729 (46.5%) men, aged 16 to 90 years (mean age = 51.68 years, SD = 17.25), with 147 (3.8%) self-identified as South Asian, and 3569 (91.7%) as Caucasian.

### Measures

#### Ethnicity

Ethnic classifications were based on 18 groupings [41]. People who self-identified as ‘White’ (English/Welsh/Scottish/Northern Irish/British), ‘White—Irish’, ‘White—Gypsy or Irish Traveller’ or ‘Any other White background’ were labelled ‘*Caucasian*’ (1), while respondents who identified themselves as ‘Indian’, ‘Pakistani’, or ‘Bangladeshi’ were labelled as ‘*South Asian*’ (0).

#### HbA1c Levels

HbA_1c_ results were based on non-fasting blood samples [40]. HbA_1c_ depicts the percentage of haemoglobin in the blood that contains glucose, over the preceding 8 to 12 weeks [19]. The HSE survey incorporated up to eight HbA_1c_ parameters including glycated haemoglobin result (%) (blood-based data), and glycated haemoglobin result (in mmol/mol) (blood data). A HbA_1c_ value of 6.5% (or 48 mmol/mol) is the recommended maximum cut-off beyond which diabetes may be diagnosed [16] (note: a value below 6.5%/48 mmol/mol does not completely rule out the possibility an individual has diabetes). HbA_1c_ values below 6% (42 mmol/mol) are considered ‘normal’, while values ranging from 6 to 6.4% (42 to 47 mmol/mol) suggests pre-diabetes. From June 2009, laboratories in the UK have been reporting HbA_1c_ results primarily in millimoles per mole rather than in percentages (%). Thus, for consistency, this study used glycated haemoglobin results calibrated in millimoles per mole as the primary outcome variable.

#### Mental Wellbeing

Positive *mental wellbeing* was the primary ‘exposure’ variable of interest, as this factor was expected to ‘explain’ (i.e. mediate) ethnic disparities in HbA_1c_ levels. Mental wellbeing was measured using the *Warwick-Edinburgh Mental Well-being Scale* (WEMWBS) [29]. Respondents are asked to read each of the 14 statements describing various feeling and thoughts, and then indicate the extent to which each item had been experienced during the past 2 weeks. Examples of items include ‘been feeling optimistic about the future’, ‘been feeling useful’, ‘been feeling relaxed’, ‘been feeling interested in other people’, ‘had energy to spare’, ‘been dealing with problems well’, ‘been thinking clearly’, and ‘been feeling good about myself’. Responses were indicated on a 5-point Likert-style scale: ‘None of the time’ (1), ‘Rarely’ (2), ‘Some of the time’ (3), ‘Often’ (4), and ‘All of the time’ (5). Responses were totaled to give a single score depicting mental wellbeing, with a higher score depicting better psychological functioning (*α* = 0.93). To obtain greater clarity regarding the role of *specific* aspects of mental wellbeing in HbA_1c_ outcomes across ethnic groups, it was decided to treat the 14 WEMWBS domains as single-item measures [42]. The use of single-item measures is justified when (a) weak or modest effect sizes are expected (there is no previous evidence suggesting a particularly strong mediating effect of mental wellbeing on ethnicity-HbA_1c_ relations), (b) scale items are highly homogeneous (*α* > 0.90) (internal consistency for the mental wellbeing items exceeded this value), (c) items are semantically redundant (due to high internal consistency), which negatively affects multiple item measures, and (d) the population is diverse (e.g. ethnic differences) [42, 43]. Moreover, the use of single-items in an ethnicity/HbA_1c_ context may have important practical implications for health care. For example, it could enable health professionals more easily identify and address *specific* elements of psychological functioning implicated in elevated HbA1c levels, within a particular ethnic group. The term ‘mental wellbeing’ is arguably too broad a concept to manipulate in the context of a health education program, or routine doctor-patient consultations.

#### Covariates

Covariates consisted of age, gender, mental wellbeing, employment earnings, biomedical factors (diabetes status, blood pressure, body mass index/BMI) and lifestyle factors (physical activity, cigarette smoking).

*Physical activity* was measured using the short version of the *International Physical Activity Questionnaire* (IPAQ) [44]. This instrument assesses physical activity across a range of domains including leisure time, yard activities (e.g. gardening), and work-related activities. The short IPAQ evaluates three activity types: walking, moderate-intensity activities, and vigorous intensity activities. Frequency (calibrated in days per week) and duration (time per day) are computed for each activity type. This data is then used to compute a total physical activity score for each respondent, for each activity type. The data was used to categorise respondents into two groups: ‘inactive, below 30 min MVPA (moderate-vigorous physical activity) per week’ (0) and ‘active, 30 min or more’ (1). *Cigarette smoking* was assessed using various items, including simply asking respondents to indicate the number of cigarettes they smoke per day.

Socioeconomic *deprivation* was based on a simple dichotomy depicting employment-related deprivation. Respondents were shown a card listing various possible sources of income, including ‘earnings from employment or self-employment’, and then asked to indicate which they received. For the purpose of this study responses to this employment-related item were coded as follows: ‘Earnings from employment or self-employment’ (1), ‘no earnings from employment or self-employment’ (0).

Several *biomarkers* were gauged. *Blood pressure* (BP) was measured using the Omron HEM 907 blood pressure monitor. The data was used to generate three distinct groups: ‘BP under 130/80’ (1), ‘BP under 140/90, but not under 130/80’ (2), ‘BP 140/90 or above’ (3). Respondents were also asked whether they had ever been told by a doctor they had high blood pressure (HBP), then dichotomised into two groups; doctor diagnosed high blood pressure (excluding pregnant), ‘yes’ (1), ‘no’ (2). Respondents also provided height and weight measurements, which were used to compute *BMI* scores (kg/m^2^), generating three groups: ‘Underweight’ (BMI < 18.5), ‘normal’ (BMI, 18.5 to <25), ‘overweight’ (BMI, 25 to <30), ‘obese’ (BMI, 30 to <40), and ‘morbidly obese’ (BMI, ≥ 40).

*Diabetes* status was gauged via multiple questions, including; whether respondents currently have, or have ever had diabetes; whether they had been told by a doctor that they had diabetes; and whether they had been told by a doctor/nurse that they had *Type* I or *Type* II diabetes. For the purposes of this study, diabetes status—‘case’ (1) versus ‘non-case’ (0)—was primarily based on the first item. This helped address sources of ambiguity where for example, a respondent may have had diabetes since childhood, but been unable to recall being informed about this by a doctor or nurse.

## Results

### Descriptive Data

Table 1 shows descriptive data for the sample. HbA_1c_ levels were, on average, significantly higher in South Asians compared with Caucasians, albeit values fell below 6% (42 mmol/mol), and hence may be considered ‘normal’ (i.e. depicting ‘good’ blood sugar control for both ethnic groups) [16]. One hundred and eighty-one Caucasians and 15 South Asians met the HbA_1c_ criterion for *diabetes* (HbA_1c_ > 48 mmol/mol). Another 319 Caucasians and 15 South Asians fitted the HbA_1c_ criterion for *pre-diabetes* (HbA_1c_ of 42 to 47 mmol/mol). South Asians were significantly (approximately 10 years) younger, had a slightly lower BMI score, and smoked fewer cigarettes, compared with Caucasians. South Asians also reported slightly better mental wellbeing albeit this group difference wasn’t statistically significant (*p* = 0.054). South Asians were more likely to receive an income from employment or self-employment. Over 90% of respondents did not currently have, or had ever had, diabetes (just over 6% were diabetes cases). Nevertheless, diabetes prevalence in South Asians was nearly twice the rate in Caucasians. There were no ethnic differences in blood pressure groupings, or BP diagnosis by a doctor.

**Table 1:** Descriptive statistics

	South Asian	Caucasian	
Age	*M* = 42.01 (SD = 14.35)	*M* = 52.48 (SD = 17.23)	*p* < 0.001
Gender			
Male	77 (52.4%)	1652 (46.3%)	n.s.
Female	70 (47.6%)	1917 (53.7%)	
Employment earning (receive earnings from employment or self-employment)			
No	27 (21.8%)	1152 (33.1%)	*p* < 0.01
Yes	97 (78.2%)	2328 (66.9%)	
Blood sugar level (glycated haemoglobin/HbA_1c_ result, in mmol/mol/%	39.87 (10.33)/5.8%	37.69 (8.31)/5.6%	*p* < 0.05
Mental wellbeing (WEMWBS total score)	52.49 (9.06)	50.93 (8.73)	n.s.
Body mass index (BMI) score	26.78 (4.46)	27.63 (5.26)	*p* < 0.05
Body mass index (BMI) groups (excluding underweight and combining obese and morbidly obese)			
Normal	48 (35.6%)	1120 (34.1%)	n.s
Overweight	60 (44.4%)	1256 (38.3%)	
Obese	27 (20%)	904 (27.6%)	
Cigarette smoking (number of cigarettes smoked a day—including non-smokers)	0.95 (3.45)	1.97 (5.37)	*p* < 0.001
Diabetes (currently have, or ever had diabetes)_			
No	131 (89.1%)	3355 (94.1%)	*p* < 0.05
Yes	16 (10.9%)	211 (5.9%)	
Blood pressure groups			
BP under 130/80	75 (60.5%)	1679 (54.1%)	n.s.
BP under 140/90 but not under 130/80	27 (21.8%)	755 (24.35)	
BP 140/90 or above	22 (17.7%)	671 (21.6%)	
Blood pressure—doctor diagnosed (excluding pregnant women)			
No	119 (81%)	2691 (75.4%)	n.s.
Yes	28 (19%)	876 (24.6%)	
Physical activity (IPAQ)			
Inactive (below 30 min Moderate/vigorous PA per week)	48 (43.6%)	929 (29.6%)	*p* < 0.01
Active (30 min moderate/vigorous PA or more per week)	62 (56.4%)	2210 (70.4%)	

Data are means (SDs) or N (%), unless otherwise stated

*n.s*. not significant

Gender (*p* = 0.147); Mental wellbeing (*p* = 0.054); Body Mass Index (*p* < 0.05); Blood pressure groups (*p* = 0.359); Blood pressure - doctor diagnosed (*p* = 0.127)

### Bootstrapping

We used an SPSS bootstrapping dialogue [45, 46] to assess the *direct* association between ethnicity (Caucasian versus South Asian) and HbA_1c_ levels, and the *indirect* relationship between these variables, mediated by the 14 mental wellbeing domains of the WEMWBS [29]. Bootstrapped sampling was set at 1000. Furthermore, effect size, Sobel normal theory test [47], and total effect data were generated. For each mediated regression model, ethnicity was specified as the ‘predictor’ (‘variable X’), HbA_1c_ was entered as the ‘outcome’, while mental wellbeing factors were analysed as the ‘mediators’. Thus, several regression pathways were examined: the association between ethnicity and dimensions of mental wellbeing (*path a*), the relationships between mental wellbeing domains and HbA_1c_ levels (*path b*), the direct association between ethnicity and HbA_1c_ levels (*path c*), and the indirect effect of ethnicity on HbA_1c_ scores, mediated by mental wellbeing dimensions (path a*b). Age, gender, mental wellbeing, employment earnings, biomedical factors (diabetes status, blood pressure, body mass index/BMI), and lifestyle factors (physical activity, cigarette smoking) were treated as covariates. Due to software constraints on the total number of mediator variables that can be tested simultaneously [45], each analysis was performed twice, initially using the first ten mental wellbeing domains, and then again using the last four dimensions. To reduce the probability of type 1 errors (‘false positives’), statistical significance for mediator effects was based on the conservative Sobel normal theory test [47].

**Fig. 1:**
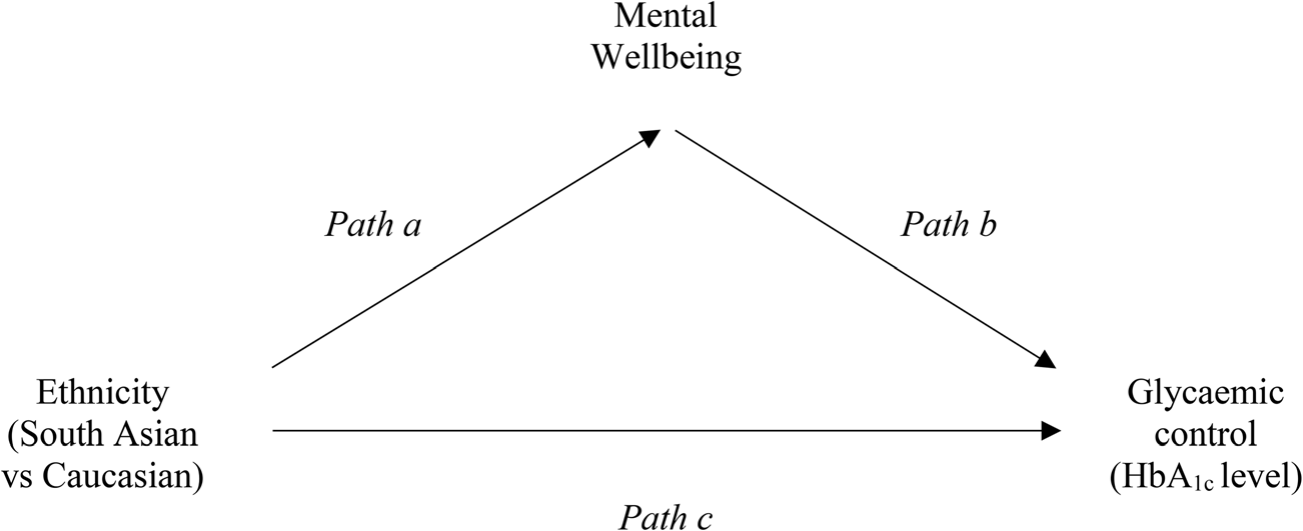
Proposed mediating effect of mental wellbeing on blood sugar control across ethnic groups

**Fig. 2:**
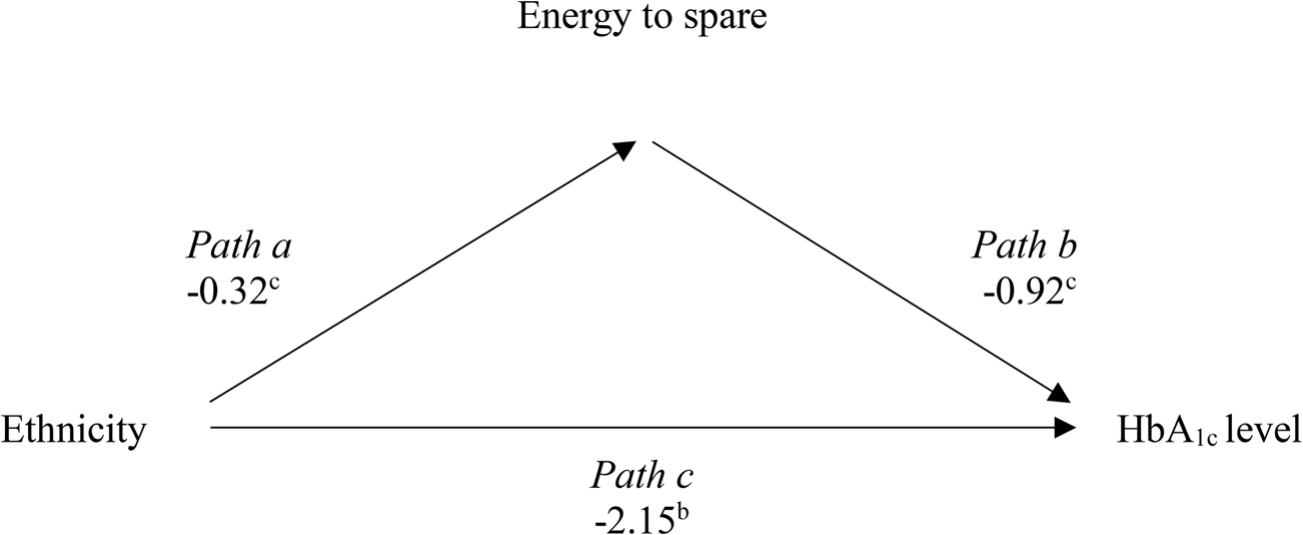
Observed mediating effect of perceived energy to spare on blood sugar control across South Asians and Caucasians. Note: ^a^(*p* < 0.05), ^b^(*p* < 0.01), ^c^(*p* < 0.001)

Bootstrapping revealed *both* direct and indirect associations (see Fig. 1) [45]. There was a significant *direct* association (*path* c) between ethnicity and HbA_1c_ (see Table 2); Caucasians obtained lower HbA_1c_ values compared with South Asians. Several domains of mental wellbeing also predicted HbA_1c_ (*path* b); HbA_1c_ levels were lower given greater perceived energy to spare, and higher optimism about the future, but less relaxation. Ethnicity predicted several aspects of mental wellbeing (*path* a), most notably perceived energy levels; Caucasians felt significantly less energetic compared to South Asians. Furthermore, the indirect effect of ethnicity on HbA_1c_, via perceived energy (*paths* a*b), was significant, based on the conservative normal theory (i.e. Sobel) test. This mediator effect accounted for 14.1% of the relationship between ethnicity and HbA_1c_, and is illustrated in Fig. 2; lower HbA_1c_ levels were associated with greater perceived energy, which in turn was more typical of South Asians.

**Table 2:** Ethnic differences in blood sugar control (HbA_1c_) with ‘energy to spare’ as the mediator, before and after adjusting for selected biopsychosocial covariates

Variables	Path a (ethnicity → energy)	Path b (energy → HbA_1c_)	Path c (ethnicity → HbA_1c_)	Path a*b or indirect effect (ethnicity → energy → HbA_1c_)	Total effect
Unadjusted	−0.32 (−0.49, −0.160)***	−0.92 (−1.29, −0.55)***	−2.15 (−3.64, −0.67)**	0.30 (0.13, 0.58)**	−1.83 (−3.32, −0.35)*
Adjusted for age (0 to 90), and gender (male = 1, female = 2), employment earnings (receiving = 1, not = 0)	−0.26 (−0.44, −0.09)**	−0.44 (−0.80, −0.07)*	−4.05 (−5.61, −2.49)***	0.11 (0.01, 0.32)****	−3.91 (−5.47, −2.36)***
Adjusted for age (0 to 90), and gender (male = 1, female = 2), employment earnings (receiving = 1, not = 0), BMI score	−0.19 (−0.37, −0.00)*	−0.29 (−0.66, 0.07)***	−4.14 (−5.72, −2.57)***	0.05 (−0.00, 0.21)	−4.05 (−5.61, −2.48)***
Adjusted for age (0 to 90), and gender (male = 1, female = 2), employment earnings (receiving = 1, not = 0), BMI score, high blood pressure (yes *=* 1, no = 0)	−0.19 (−0.38, −0.01)*	−0.20 (−0.57, 0.15)	−4.05 (−5.61, −2.48)***	0.04 (−0.02, 0.17)	−3.97 (−5.53, −2.41)***
Adjusted for age (0 to 90), and gender (male = 1, female = 2), employment earnings (receiving = 1, not = 0), BMI score, high blood pressure (yes = 1, no = 0), diabetes status (had = 1, never had = 0)	−0.21 (−0.39, −0.02)*	−0.04 (−0.35, 0.26)	−2.74 (−4.06, −1.41)***	0.00 (−0.05, 0.11)	−2.70 (−4.02, −1.38)***
Adjusted for age (0 to 90), and gender (male = 1, female = 2), employment earnings (receiving = 1, not = 0), BMI score, high blood pressure (yes = 1, no = 0), diabetes status (had = 1, never had = 0), cigarette smoking (number smoked), physical activity (IPAQ ‘active’ = 1, ‘not active’ = 0)	0.24 (−0.44, −0.04)*	−0.04 (−0.36, 0.28)	−2.27 (−3.72, −0.83)**	0.01 (−0.07, 0.12)	−2.24 (−3.68, −0.81)**

The table does not include the *direct* effect of variable *X* (ethnicity) on variable *Y* (HbA_1c_) unadjusted for variance attributable to the mediator (perceived ‘energy to spare’)

*(*p* < 0.05), **(*p* < 0.01), ***(*p* < 0.001), ****p > 0.05 (indirect effect is significant based on CI’s, albeit conservative Sobel test not significant)

To ascertain the robustness of observed associations bootstrapping was repeated while adjusting for various covariates. Adjustment for demographic factors (age, gender, deprivation) slightly attenuated, but did not abolish the mediator effect. Furthermore, the direct association between ethnicity and HbA_1c_ remained significant. However, the mediator effect was no longer significant after adjusting for biomedical markers, specifically BMI. Accounting for biological factors together (BMI, diabetes status, blood pressure) markedly increased the percentage of HbA_1c_ variance explained by the total model (to 44%), but failed to negate the significant effect of ethnicity on HbA_1c_. All biomarkers significantly predicted HbA_1c_ having diabetes, higher blood pressure, and a higher BMI score were associated with higher HbA_1c_ values. Adjusting for cigarette smoking and levels of physical activity had little effect on the direct relationship between ethnicity and HbA_1c_.

Finally, to further explore the role of mental wellbeing in a clinical context, additional bootstrapping analysis was performed using only respondents with *pre-diabetes* (HbA_1c_ of 42 to 47 mmol/mol) or *diabetes* (HbA_1c_ > 48 mmol/mol). It was decided to combine the pre-diabetes and diabetes groups due to the very small number of South Asian cases. As before, bootstrapping examined the mediating effects of the 14 mental wellbeing dimensions on ethnic disparities in HbA_1c_ values. No mediating effects emerged. Furthermore, the total effect and direct effect models were both non-significant (*p* > 0.05), albeit one dimension of mental wellbeing—feeling *cheerful*—was associated with HbA_1c_ outcomes: more cheerful patients had significantly lower HbA_1c_ levels.

## Discussion

The aim of this study was to assess the degree to which positive mental wellbeing affects ethnic differences in HbA_1c_ outcomes. Some evidence for mediation emerged: perceived energy levels partly confounded the relationship between ethnicity and HbA_1c_ levels. Furthermore, ethnicity and several positive psychological dispositions directly predicted glycaemic control. HbA_1c_ values were lower amongst Caucasians. While some research has found no disparity between South Asians and Caucasians in HbA_1c_ levels [12], the present findings support previous evidence indicating poorer HbA_1c_ values in South Asians compared with Caucasians [13]. Furthermore, the current findings show that mental wellbeing concerning perceived vigour is important in understanding ethnic differences in glycaemic control.

Previous studies have found HbA_1c_ differentials between Caucasians and South Asians [21–23]. For example, one study found higher HbA_1c_ outcomes in the South Asians patients (6.11 ± 0.58%) compared to Caucasians (5.90 ± 0.40%) [21]. The present findings support previous observations. South Asians may experience poorer glycaemic control for a number of cultural and religious reasons, including unhealthy dietary norms, language discordance that hampers communication with health professionals, and misunderstanding and misinterpretation of self-management guidelines [14]. For example, compared to Caucasians, South Asians may derive a higher proportion of their food energy from fat and saturated fat, a situation compounded by use of unhealthy cooking methods such as deep fat frying [48]. Many South Asians consume foods considered detrimental to glycaemic control, due to a perception of such foods as ‘strength giving’, and/or strong cultural pressures to eat such foods together (e.g. with family members) [48]. There may also be a tendency to view diabetes as an inevitable act of divine intervention (e.g. ‘Gods will’), or hereditary, and/or to rely on Ayurveda and folk herbal treatments [49]. Nevertheless, it should be emphasised that South Asians are a very diverse group, both in a cultural (e.g. cooking styles) and religious (e.g. Hinduism, Islam) sense. Thus, the cultural explanations offered thus far don’t necessarily apply across subgroups. Furthermore, blood sugar control in this study was actually ‘normal’ for both ethnic groups, with HbA_1c_ group means falling well below the cut-offs for pre-diabetes and diabetes (HbA_1c_ < 42 mmol/mol) [16].

While previous research implicates mental wellbeing in HbA_1c_ levels [30, 31], this is the first study to assess mental wellbeing as a *mediating* factor that partly accounts for ethnic differentials in HbA_1c_ values. South Asians reported higher perceived energy levels, and this mind-set was associated with lower HbA_1c_ levels. Thus, perceived energy may play a protective glycaemic role for South Asians, in terms of better glycaemic control and hence reduced diabetes risk. There may be cultural undertones, whereby South Asians for example enjoy the various benefits of an extended family system that help to reduce physical exhaustion (e.g. help with childcare, preparing meals, house chores). These benefits may be less available to Caucasians, assuming a predominantly nuclear family system. Having more energy could mean a greater capacity to engage in self-care behaviours critical to glycaemic control, most notably physical activity. Thus, the excess HbA_1c_ levels in South Asians, relative to Caucasians, may be partly attributable to less perceived vigour in some South Asian individuals.

It should be noted that this mediator effect wasn’t replicated when bootstrapping was performed using only pre-diabetes and diabetes cases. Furthermore, the effect accounted for a modest proportion (<15%) of the variance in HbA_1c_ levels. This suggests the South Asian deficit (i.e. higher HbA_1c_ value) in blood sugar control is largely attributable to other factors, possibly unrelated to mental wellbeing, irrespective of clinical diagnosis. Moreover, the mediator effect was negated after accounting for BMI, suggesting body weight may play a key role in this context. Research suggests energy levels are related to body weight, particularly in men [50]. Body weight, in turn, has been associated with ethnic differences in diabetes risk [6]. Evidence also suggests that, compared to Caucasians, some ethnic minority groups are more susceptible to *chronic fatigue syndrome*, a condition typified by unexplained exhaustion [51]. Research has found that chronic fatigue is highly prevalent in both Type 1 and Type 2 diabetes sufferers [52]. However, it is unclear to what extent this condition accounts for the mediator effect observed here, especially given that Caucasians felt *less* energetic, and the association between chronic fatigue and HbA_1c_ is unclear [52]. It is possible the elevated HbA_1c_ levels amongst South Asians found here, and reported in the literature [21], should actually be higher if not for the dampening effect of greater perceived energy, which may help facilitate the performance of self-care activities essential for glycaemic control (e.g. physical activity).

Accounting for biomedical factors significantly increased the proportion of HbA_1c_ variance explained. This is unremarkable given the strong associations between biological factors, notably BMI and diabetes status, and blood sugar control [35]. Taken together the emerging patterns suggest a plausible scenario, whereby ethnicity relates to HbA_1c_ levels, both directly as previously reported in the literature [21–23], and indirectly through perceived energy levels. This latter observation is the most important finding from this study. While energy levels are related to body weight [53], and have physiological underpinnings, it is the *perception* of lack of energy that is critical here. South Asians, overall, appear to have a more positive mind-set regarding personal vivacity, with potentially favourable implications for glycaemic control.

### Limitations

This study has a number of limitations. Firstly, there was a notable reduction in sample size as each covariate (or set of covariates) was controlled for, due to missing data. Accounting for the final set of covariates (lifestyle factors) reduced the sample to just over 2911, possibly increasing the risk of *type* II errors. The small number of South Asian participants (relative to the number of Caucasian respondents) is a particular concern; it is possible the results may not generalise to the wider South Asian population. Nevertheless, it is important to note that the data used here is based on a representative (stratified) sample of UK families and hence may roughly reflect ethnic demographic proportions and diabetes prevalence in the general UK population [40]. Another limitation is selectivity in the covariate adjustments. Numerous biopsychosocial factors may affect HbA_1c_ results [16]. For example, genetic characteristics, cardiovascular disease, and a range of illness-related factors have been implicated in HbA_1c_ levels [16]. Insulin resistance has been identified as a key factor in the South Asian surplus diabetes risk [6]. Attempting to control for every conceivable covariate is impractical, so a selection of the more salient covariates was assessed here (e.g. obesity/BMI, diabetes status). Nevertheless, it is conceivable the direct effects of ethnicity observed here may be negated by uncontrolled covariates (e.g. insulin resistance). Finally, interpretation of ethnic differences may be confounded by the failure to distinguish between different South Asian communities (e.g. Indian, Bangladeshi, and Pakistani). South Asians are a culturally and religiously diverse group [48], a diversity that extends to diabetes-related outcomes. For example, diabetes risk is significantly higher in South Asians of Indian or African Asian descent, compared to those with a Pakistani or Bangladeshi background [7].

### Conclusions

This study confirms previously reported ethnic disparities in glycaemic control, with South Asians experiencing higher HbA_1c_ outcomes compared to Caucasians. The findings build on existing literature by highlighting the mediating effect of *perceived* energy in this context. Hitherto previous research on ethnic differences in glycaemic control have not examined the contribution of positive mental wellbeing. The current results suggest ethnic inequalities in glycaemic control are partly explained by how energetic people perceive themselves to be, albeit this effect is modest and negated after accounting for BMI and other covariates. Perceived energy may serve a protective glycaemic function in South Asians, as this perception was associated with lower HbA_1c_ levels, and more pronounced amongst South Asians compared to Caucasians. However, ethnic differences in HbA_1c_ transcend biopsychosocial factors including diabetes status and were only marginally explained by the mediating effect of perceived energy. The present findings justify concerns regarding an elevated diabetes risk in South Asians relative to Caucasians. Poorer glycaemic control in the former group may be dampened by individual differences in perceived energy levels, based on the present data. This mediator effect necessitates further analysis of the possible role of conditions such as chronic fatigue syndrome in the higher HbA_1c_ levels amongst South Asians.
